# Quality of life of pediatric patients operated for pilonidal sinus disease

**DOI:** 10.1007/s00431-022-04678-3

**Published:** 2022-11-08

**Authors:** Ciro Esposito, Benedetta Lepore, Mariapina Cerulo, Rachele Borgogni, Fulvia Del Conte, Vincenzo Coppola, Claudia Di Mento, Roberto Carulli, Roberto Cardone, Giuseppe Cortese, Giorgia Esposito, Maria Escolino

**Affiliations:** 1 Pediatric Surgery Unit, Department of Translational Medical Science, University of Naples “Federico II”, Via S. Pansini 5, 80131 Naples, Italy; 2 Pediatric Anesthesiology Unit, Department of Translational Medical Science, University of Naples “Federico II”, Via S. Pansini 5, 80131 Naples, Italy

**Keywords:** Pilonidal sinus disease, PSD, PEPSIT, Children

## Abstract

Quality of life (QOL) outcome is an ideal method for determining the efficacy of a surgical treatment. In children operated for pilonidal sinus disease (PSD), open procedures imply prolonged wound care, significant morbidity, and high recurrence rates. Endoscopic treatment (PEPSIT) overcomes these limitations. We report our experience in the management of PSD to evaluate the QOL of patients undergoing open and endoscopic treatment. The records of 177 patients undergoing surgery for PSD from 2008 to 2021 were retrospectively reviewed. Twenty patients were operated with open surgery (G1) and 157 with PEPSIT (G2). We analyzed QOL through the following criteria: hospital stay (HS), healing time (HT), return to sport (RTSp), return to school (RTSc), resumption of social life (RSL), and recurrence rate and reoperation (RRR). Moreover, we used Pediatric Quality of Life Enjoyment and Satisfaction Questionnaire (PQ-LES-Q) for a more subjective evaluation of life satisfaction. We found significant differences in all the analyzed criteria: HS varied from 3 to 7 days in G1 and from 1 to 2 days in G2; HT from 40 to 75 days in G1 while from 20 to 41 days in G2; RTSp from 50 to 80 days in G1 while from 7 to 21 days in G2; RTSc from 9 to 15 days in G1 while from 2 to 4 days in G2; RSL from 13 to 20 days in G1 while from 2 to 5 days in G2; RRR was 25% in G1 and 4.4% in G2.

*Conclusion*: Endoscopic treatment (PEPSIT) significantly improves the quality of life of patients operated for PSD. Compared to open surgery, PEPSIT presents shorter hospital stay, faster healing time, return to sport activities, return to school and resumption of a normal social life, and lower rates of recurrence and reoperation. In addition, PQ-LES-Q demonstrated a good overall quality of life and life satisfaction. Further prospective studies should be obtained to consider PEPSIT as the gold standard for the treatment of PSD in pediatric patients.

**Table:** 

**What is Known:**
• *Many techniques have been proposed in the last 20 years for the surgical treatment of PSD.*
• *PEPSIT is showing promising results in terms of safety and long-term efficacy.*
**What is New:**
• *The main impact in QOL of patients operated with PEPSIT is on their daily activity, including a shorter hospital stay, faster healing time, return to sport activities, return to school and resumption of a normal social life, lower rates of recurrence and reoperation.*
• *After PEPSIT, children maintain a satisfactory quality of life according to the analysis of PQ-LES-Q*

## Introduction

Pilonidal sinus disease (PSD) is an acquired inflammatory condition affecting the gluteal cleft, with a reported incidence among children of 26:100,000 [1–4]. PSD mostly affects adolescents and young adults with a peak incidence between 13 and 22 years [5–8].

Especially in this age group, the symptoms of PSD can be debilitating, both physically and socially, and surgical therapy is often required. Many techniques have been proposed in the last 20 years for the surgical treatment of PSD. According to the literature, open surgery with a wide excision of cutaneous and subcutaneous tissue of the affected area until the presacral fascia is often the technique of choice [9]. However, open procedure requires general anesthesia and is associated with high morbidity, including wound complications, prolonged healing time, and high recurrence rate [10].

In recent years, several papers about pediatric endoscopic treatment of PSD (PEPSIT) have been published, showing promising results on its safety and long-term efficacy. PEPSIT has been correlated with less post-operative discomfort for the patients and lower recurrence rates [11–13].

Endoscopic treatment of pilonidal sinus disease (EPSIT) was firstly described by Meinero in 2014 in the adult population [14]. In 2015, our group modified this approach [5], creating a new structured multistep protocol that implies pre- and post-operative laser epilation, PEPSIT, and accurate post-operative wound management with oxygen-enriched oil-based gel dressing [15, 16].

Quality of life (QOL) outcome is the most adopted evaluation method for determining the efficacy of a surgical therapy in operated patients [17, 18], while Pediatric Quality of Life Enjoyment and Satisfaction Questionnaire (PQ-LES-Q) represents a valid assessment tool to evaluate the quality of life and life satisfaction in adolescents [19].

In this study, we analyzed our experience during the last 14 years on the management of this disease with the aim to evaluate the QOL after open and endoscopic treatment.

## Materials and methods

We retrospectively analyzed the data of pediatric patients affected by pilonidal sinus disease (PSD) operated in our center from January 2008 to October 2021 with open and endoscopic approaches.

During this period, a total of 177 pediatric patients with PSD were treated surgically. There were 113 boys and 64 girls, with a median age of 15.5 years (13–17). Patient characteristics are showed in Table 1. All patients had a minimum follow-up of 6 months. All patients attended the outpatient department at 1 week, 1 month, 3 months, 6 months, and then every year post-operatively.

**Table 1:** Patients characteristics

Parameter	G1 (*n* = 20)	G2 (*n* = 157)	*p* value
*M*/*F*, *n*/*n*	7/13	69/88	
Median patient age, years (range)	16 (13–17)	15.4 (13–17)	0.01
Disease characteristics	Mixed	Mixed	
Type of anesthesia	General	Subarachnoid	n.a
Analgesia, days (range)	5 (3–7)	0 (0–3)	< 0.01
Drainage	Required	Not required	n.a
Laser session	Not applied	Applied	n.a
Period of study (years)	9	5	n.a
Median patients/year (range)	2.2 (0–5)	33 (25–47)	n.a
Follow-up, months (range)	24 (20–36)	28 (15–60)	< 0.01

Of 177 patients analyzed, 20 were operated using open approach (G1) in a 9-year period (2008–2016) with a median of 2.2 patients operated per year (0–5) and 157 patients underwent endoscopic approach with PEPSIT (G2) in a 5-year period (2017–2021) with a median of 33 patients per year (25–47) (Table 1).

G1 patients received general anesthesia. The surgery involved a complete resection of PSD with a primary closure of the defect using separated stitches. A drainage was left in place for 24–48 h. Prone decubitus for 3–4 days after surgery was maintained in the post-operative period.

G2 patients received 2–3 pre-operative sessions of pulse-dye laser epilation at 4–6-week interval before surgery. Subarachnoid spinal anesthesia was performed before surgery. During PEPSIT procedure, a fistuloscope was introduced through the PSD orifice(s), hairs removed with endoscopic forceps, cavity debrided with the endobrush, and ablated with monopolar electrode. Normal decubitus was maintained in the post-operative period.

In the post-operative period, G1 patients had a mean analgesic need of 4 days: paracetamol and NSAIDS were the drugs of choice, given at 6- to 8 h interval. The analgesic requirement in G2 was absent for most patients.

**Fig. 1:**
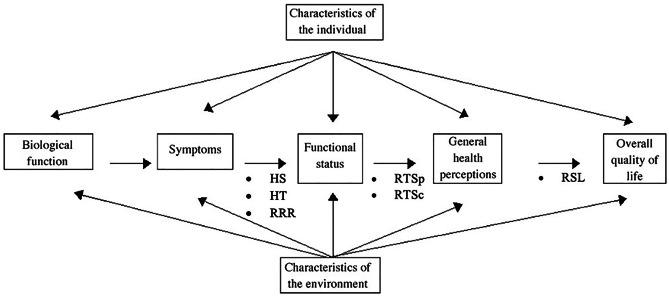
Variables proposed to influence health-related quality of life according to Ferrans et al. [17]. Bullet lists did not appear in the original model and were added exclusively for this study

The quality of life (QOL) of both groups was analyzed to evaluate long-term outcomes of the two techniques. Ferrans and colleagues’ revision of the Wilson and Cleary’s model of health-related QOL was selected for the organizing framework guiding this study and modified by our group for pediatric patients (Fig. 1). We focused on the following 6 parameters: hospital stay (HS), healing time (HT), return to sport (RTSp), return to school (RTSc), resumption to social life (RSL), and recurrence rate and reoperation (RRR). All patients were interviewed during their follow-up visits.

G2 patients were also evaluated using PQ-LES-Q 1 week after discharge from the hospital.

Statistical analysis was carried through Student’s *t*-test. Significance was defined as *p* value < 0.05. For the *t*-test to be applied, G1 and G2 samples need to fulfill the hypotheses of normality, which have been correctly verified.

The study received the appropriate Institute Review Board (IRB) approval. Informed consent to participate in the study was obtained from all participants (or their parent or legal guardian in the case of children under 16).

## Results

Table 2 shows quality of life (QOL) outcomes of the two groups of study. G1 reported a median HS of 5 days (3–7), which was significantly longer than that reported by G2 (median of 36 h, interval of 1–3 days) (*p* = 0.002). An important parameter justifying this difference is the prone decubitus that G1 patients had to maintain for 3–4 days, with respect to G2 patients undergoing PEPSIT procedure, who were placed in a normal position immediately after surgery. Concerning HT, results showed a variation from 40 to 75 days (median 56 days) for G1 patients, while G2 patients experienced an average healing time of 28 days (20–41) (*p* < 0.00001). This important difference in favor of G2 group is related to the fact that medications after open surgery needed to be performed inside the hospital, every other day until 3–4 weeks post-operatively. On the contrary, PEPSIT procedure allowed G2 patients to be discharged and perform wound management at home, with a simple disinfection and injection of oxygen-enriched oil-based gel in PSD orifice(s), easily performed by parents/caregivers.

**Table 2:** QOL outcomes

Parameter	G1 (*n* 20)	G2 (*n* 157)	*p* value
HS, days (range)	4.2 (3–7)	1.5 (1–3)	0.002
HT, days (range)	56 (40–75)	28 (20–41)	< 0.00001
RTSp, days (range)	60 (50–80)	14 (7–21)	< 0.00001
RTSc, days (range)	13 (9–15)	3 (2–4)	< 0.00001
RSL, days (range)	16 (13–20)	3 (2–5)	< 0.00001
RRR, overall *n* (%)	5 (25)	7 (4.4)	< 0.00001

*HS* Hospital Stay, *HT* Healing Time, *RTSp* Return to Sport, *RTSc* Return to School, *RSL* Return to Social Life, *RRR* Recurrence Rate and Reoperations

The RRR parameter was considered for PSD patients which, after complete wound healing and with no reported history of trauma in the coccygeal region, presented a new sinus, regrowth of hair in the sinus orifice, or discharge of purulent material, thus requiring reintervention. In our study, RRR was 25% in G1 and 4.4% in G2 (*p* < 0.00001).

As regards the “non-clinical” parameters, we found a statistically significant difference for each of them. RTSp varied from 50 to 80 days (median 60 days) in G1 compared to G2 patients, which experienced an average return to sport after 14 days (range 7–21) (*p* < 0.00001).

RTSc was on average of 13 days (range 9–15) in G1 and of 3 days (range 2–4) in G2 (*p* < 0.00001). In fact, open surgery delayed the return to school time because children found difficult to seat down for a long time (4–6 h) every morning at school.

RSL varied from 13 to 20 days (median 16 days) in G1 and from 2 to 5 days (median 3 days) in G2 (*p* < 0.00001).

G2 patients were also evaluated using PQ-LES-Q (Table 3).

**Table 3:** PQ-LES-Q outcomes

Over the past week, how have things been with…	G2 (*n* 157)
Your health	4 (2–5)
Your mood or feelings	5 (4–5)
School or learning	5 (4–5)
Helping out at home	4 (2–5)
Getting along with friends	5 (3–5)
Getting along with your family	5 (3–5)
Play or free time	4 (2–5)
Getting things done	5 (3–5)
Your love or affection	5 (3–5)
Getting or buying things	5 (2–5)
The place you live at	5 (3–5)
Paying attention	4 (3–5)
Your energy level	4 (2–5)
Feelings about yourself	4 (3–5)
Overall, how has your life been?	5 (2–5)

Each item can be rated from 1 (very poor) to 5 (very good)

In addition, G2 patients experienced a good overall life satisfaction according to PQ-LES-Q.

## Discussion

Pilonidal sinus disease (PSD) is a common inflammatory disease of the sacrococcygeal region. According to the literature, no consensus exists on the preferred surgical technique to treat this condition. Open surgery, which involves a wide excision of cutaneous and subcutaneous tissue of the affected area until the presacral fascia [9], is often the technique of choice worldwide. However, open procedure requires general anesthesia and is associated with high morbidity, including wound complications, prolonged healing time, and high recurrence rates [10]. Moreover, patients experience an important limitation of physical and school activities, not only in terms of time.

Gips technique [6] was the first minimally invasive surgery technique described for the treatment of PSD, consisting in hair removal through the orifice(s) of the fistula using a special device.

In adults, endoscopic pilonidal sinus treatment (EPSiT) was firstly described by Meinero et al. [14] in 2014, transforming completely the management of patients with PSD. The first reported study in the pediatric population was illustrated by our group in 2017 [4], describing a series of 15 patients undergoing EPSiT, with complete removal of the sinus by ablation after the introduction of a fistuloscope, which left a minimal surgical wound upon insertion.

Quality of life (QOL) studies are considered a good model for the evaluations of medical and surgical treatment outcomes and impact on patients’ lives [17–21]. Ferrans et al.’s revision of the Wilson and Cleary’s model of health-related QOL [17] has frequently been adopted for oncology [22–24] but can be equally applied to evaluate the outcome after surgery [20].

To our knowledge, there is no existing paper focused on QOL of pediatric patients after surgery for PSD. For this reason, we decided to retrospectively analyze our experience with open and endoscopic approach for PSD management, to evaluate the QOL of patients after two different surgical procedures: open surgery and PEPSIT.

We selected Ferrans and colleagues’ revision of the Wilson and Cleary’s model of health-related QOL for the organizing framework guiding this study and we modified it for pediatric patients (Fig. 1). We focused our attention on the following six parameters: hospital stay (HS), healing time (HT), return to sport (RTSp), return to school (RTSc), resumption to social life (RSL), and recurrence rate and reoperation (RRR).

Our analysis was further implemented when we started to perform PEPSIT. In particular, we submitted the PQ-LES-Q [19] to patients operated with this technique 1 week after surgery, in order to assess their overall quality of life and life satisfaction.

The results of our study show that PEPSIT has many advantages with respect to the traditional open surgery technique for PSD. In fact, the open approach has important consequences on the post-operative period, with a median healing time of 56 days, which is doubled compared to PEPSIT patients.

Medications after open surgery need to be performed always inside the hospital, every other day until 3–4 week post-operatively, because they included the periodic introduction of an antibiotic-filled gauze into the cavity until healing by secondary intention. On the contrary, PEPSIT allows patients to be discharged and perform wound management at home, with a simple disinfection and injection of oxygen-enriched oil-based gel in PSD orifice(s), easily performed by parents/caregivers.

The main impact in QOL of patients operated with PEPSIT is on their daily activity: in fact, they refer little to any limitation after surgery compared to patients undergoing the open procedure which, instead, present limitations in physical activities for almost 2 months post-operatively.

Hospital stay is on average 5 days longer after open surgery: this is not only due to the invasiveness of the surgical procedure, but also to the fact that children need to assume a prone position for 2–3 days post-operatively and have a drainage in place for 24–48 h after surgery. On the contrary, PEPSIT allows a hospitalization that is on average 36 h long, in some cases even a 1-day surgery.

Another important aspect characterizing QOL of patients after surgery is the rate of recurrence and reoperation (RRR), that in our series reached 25% after open surgery (4 recurrences and reoperations on a total of 20 patients) and 3.4% after PEPSIT (5 recurrences and reoperations on a total of 157 patients).

After open surgery, patients also reported important discomfort in the post-operative period for the pain suffered, for the very long healing time with a late return to sport, social activities, and school.

The analysis of PQ-LES-Q could give a picture of the overall life satisfaction of patients operated with PEPSIT. Children answer to the questions with a score that ranged from 4 to 5, where 5 is the highest possible score. This means that, after PEPSIT, children maintained a satisfactory quality of life.

Based on the results of our study, endoscopic treatment (PEPSIT) dramatically improves the quality of life of patients operated for PSD compared with open surgery. PEPSIT presents several advantages compared with open surgery, including a shorter hospital stay, faster healing time, return to sport activities, return to school and resumption of a normal social life, and lower rates of recurrence and reoperation.

Considering the important consequences that social life, school attendance, and sport activities have on adolescents, our study emphasizes how children with PSD could benefit from the endoscopic treatment instead that from open surgery and how this could minimize long-term negative effects that the latter may have on their life.

Even if this study has some limitations, as the difference in number of the two groups and the absence of a comparative PQ-LES-Q between the two groups, we strongly advice the use of PEPSIT for the treatment of PSD in pediatric patients. Further prospective studies should be obtained to consider PEPSIT as the gold standard for the treatment of PSD in pediatric patients.

## Data Availability

The authors confirm that the data supporting the findings of this study are available within the article.

## Code availability

N/a.
